# Combined risk factors for melanoma in a Mediterranean population

**DOI:** 10.1054/bjoc.2001.2029

**Published:** 2001-09-01

**Authors:** M T Landi, A Baccarelli, D Calista, A Pesatori, T Fears, M A Tucker, G Landi

**Affiliations:** Genetic Epidemiology Branch, Division of Cancer Epidemiology and Genetics, National Cancer Institute, 6120 Executive Blvd, MD, Bethesda, 20892-7236, USA; EPOCA, Epidemiology Research Center, University of Milan, Via S. Barnaba 8, Milano, 20122, Italy; Dermatology Unit, Bufalini Hospital, Viale Ghirotti 286, Cesena, 47023, Italy; Biostatistics Branch, Division of Cancer Epidemiology and Genetics, National Cancer Institute, 6120 Executive Blvd, Bethesda, MD, 20892-7236, USA

**Keywords:** melanoma, dysplastic nevi, Mediterranean populations, pigmentation, melanoma thickness, risk factors

## Abstract

A case–control study of non-familial melanoma including 183 incident cases and 179 controls was conducted in North-Eastern Italy to identify important risk factors and determine how combination of these affects risk in a Mediterranean population. Presence of dysplastic nevi (OR = 4.2, 95% CI = 2.4–7.4), low propensity to tan (OR = 2.4, 95% CI = 1.1–5.0), light eye (OR = 2.4, 95% CI = 1.1–5.2), and light skin colour (OR = 4.1, 95% CI = 1.4–12.1) were significantly associated with melanoma risk after adjustment for age, gender and pigmentation characteristics. A chart which identifies melanoma risk associated with combinations of these factors is presented; it can be used to identify subjects who would most benefit from preventive measures in Mediterranean populations. According to the combination of these factors, a relative risk range from 1 to 98.5 was found. Light skin colour, high number of sunburns with blistering, and low propensity to tan were significantly associated with melanoma thickness, possibly indicating that individuals with these characteristics underestimate their risk and seek attention when their lesion is already advanced. © 2001 Cancer Research Campaign


*British Journal of Cancer* (2001) **85**(9), 1304–1310
© 2001 Cancer Research Campaign
doi: 10.1054/ bjoc.2001.2029, available online at http://www.idealibrary.com on IDEAL
http://www.bjcancer.com

### Combined risk factors for melanoma in a Mediterranean population

**MT Landi[1], A Baccarelli[1,2], D Calista[3], A Pesatori[2], T Fears[4], MA Tucker[1] and G Landi[3]**

[1]Genetic Epidemiology Branch, Division of Cancer Epidemiology and Genetics, National Cancer Institute, 6120 Executive Blvd, Bethesda, MD 20892-7236, USA; [2]EPOCA, Epidemiology Research Center, University of Milan, Via S. Barnaba 8, 20122 Milano, Italy; [3]Dermatology Unit, Bufalini Hospital, Viale Ghirotti 286, 47023 Cesena, Italy; [4]Biostatistics Branch, Division of Cancer Epidemiology and Genetics, National Cancer Institute, 6120 Executive Blvd, Bethesda, MD 20892-7236, USA

**Summary** A case–control study of non-familial melanoma including 183 incident cases and 179 controls was conducted in North-Eastern Italy to identify important risk factors and determine how combination of these affects risk in a Mediterranean population. Presence of dysplastic nevi (OR = 4.2, 95% CI = 2.4–7.4), low propensity to tan (OR = 2.4, 95% CI = 1.1–5.0), light eye (OR = 2.4, 95% CI = 1.1–5.2), and light skin colour (OR = 4.1, 95% CI = 1.4–12.1) were significantly associated with melanoma risk after adjustment for age, gender and pigmentation characteristics. A chart which identifies melanoma risk associated with combinations of these factors is presented; it can be used to identify subjects who would most benefit from preventive measures in Mediterranean populations. According to the combination of these factors, a relative risk range from 1 to 98.5 was found. Light skin colour, high number of sunburns with blistering, and low propensity to tan were significantly associated with melanoma thickness, possibly indicating that individuals with these characteristics underestimate their risk and seek attention when their lesion is already advanced. © 2001 Cancer Research Campaign

**Keywords**: melanoma; dysplastic nevi; Mediterranean populations; pigmentation; melanoma thickness; risk factors

Though Mediterranean populations have been considered at low risk of cutaneous malignant melanoma (CMM) because of their relatively dark complexion, CMM incidence rates are steadily increasing in Italy, Spain, France and former Yugoslavia (Muir et al, 1987; Parkin et al, 1992).

According to the estimates for the year 2000 provided by the International Agency for Research on Cancer (Ferlay et al, 2001), the Italian national age-adjusted incidence of CMM was 4.6 cases/100 000 person-years for males and 5.5 cases/100 000 person-years for females.

The number of nevi and presence of atypical or dysplastic nevi (DN) are powerful predictors of melanoma risk (Tucker et al, 1997) even though melanoma does not always arise in pre-existing nevi. Other risk factors include skin sensitivity to sun, freckling, history of sunburns, intermittent sun exposure, and fair hair, eye, or skin colours (Bliss et al, 1995; Tucker et al, 1997). In addition, family history of melanoma is a strong risk factor for approximately 2.5–18% of melanoma patients (Greene and Fraumeni, 1979; Goldstein and Tucker, 1995; Calista et al, 2000).

These factors have been identified in populations with a large number of fair-skinned individuals. A few studies published on Mediterranean populations (Cristofolini et al, 1987; Zanetti et al, 1988; Carli et al, 1995; Rodenas et al, 1996; Espinosa Arranz et al, 1999; Naldi et al, 2000a) suggest that they share similar risk factors, but the distribution and relative weight of these factors may vary. Our study aimed at identifying the strongest risk factors for non-familial melanoma in an Italian area in which incidence has increased from 5.0 to 10.0 cases/100 000 person-years in males, and from 9.0 to 13.1 in females between 1986 and 1997 (Vinceti et al, 1999) and at determining how the combination of these factors contributes to CMM risk.

### SUBJECTS AND METHODS

We recruited newly diagnosed incident CMM cases of any stage ($n$ = 183) between December 1994 and January 1999 at the Dermatology Unit of Maurizio Bufalini Hospital in Cesena, Italy. This Dermatology Unit serves as a referral point for the regions of Southern Emilia-Romagna and Northern Marche, a population of nearly one million people. The Hospital's Ethical Committee approved the study and all participants signed an informed consent. The patients referred to the clinic were representative of patients in the entire area (Calista et al, 2000). Cases were enrolled after surgical removal of the lesion and pathological confirmation, but prior to chemotherapy or radiation therapy. Controls were spouses or close friends of the cancer cases ($n$ = 134), outpatients referred to the hospital due to small accidental trauma ($n$ = 14), and healthy volunteers from the Bufalini Hospital personnel ($n$ = 31). All cases and control subjects were from the described catchment area. 5 subjects (3 cases and 2 controls) had a family history of melanoma and were excluded from the analysis. Study subjects included 87 male and 96 female cases with 89 male and 90 female controls. The age range was 17–77 years. Controls were frequency-matched to cases by age and gender. Approximately 95% of cases and 83% of controls agreed to participate in the study.

One dermatologist (DC) performed all skin examinations of the entire body, except the genital area. Multiple lightly pigmented macular lesions, commonly present on the face, upper back and arms were defined as freckles. We classified the frequency of

*Received 2 May 2001*
*Revised 19 June 2001*
*Accepted 21 June 2001*

*Correspondence to:* MT Landi freckles by comparison with drawings describing patterns of distribution on a 6-scale category. Nevi were pigmented macules or papules greater than 2 mm in diameter, and did not include freckles, lentigines, keratoses, and other pigmented lesions. A dysplastic nevus had to be ≥ 5mm, be predominantly flat, and have at least 2 of the following criteria: variable pigmentation, indistinct borders, and irregular outline (Landi et al, 1999). One oncologist (MAT) assessed all DN diagnoses and nevi number from photographs of subjects' back, blind to case–control status. Diagnosis and nevi numbers reported throughout the paper are based on the oncologist review only. 60 subjects (37 cases and 23 controls) could not be classified as with DN or with no DN because of age, or poor quality of photographs. Since the distribution of these subjects with uncertain DN classification differed by CMM status, we used a 3-category variable (DN, uncertain DN status, no DN) in the analyses. The dermatologist (DC) assessed the skin colour of the inner part of the subjects' upper arm using a 3-category scale, i.e., dark/olive, medium, light; the eye colour using a 9-category scale, i.e., black, dark brown, light brown, brown-green, green, blue-green, dark blue, light blue, grey; and the hair colour using a 6-category scale, i.e., black, dark brown, light brown, reddish brown, blond, red (Cristofolini et al, 1987, modified).

Trained interviewers administered a pilot-tested questionnaire to cases and controls including questions on lifetime residential history, ultraviolet (UV) exposure, medical and family history of cancer and other diseases, skin reaction to sun, and sunscreen use. We recorded the latitude, altitude and proximity to the sea for each residence of at least 6 months. We calculated an index of UVB exposure which takes into account latitude and altitude of each town using the following formula: UVB index = $(\exp(15.545 - (0.039 \times \text{latitude}) + (0.0001038 \times \text{altitude}))/10\,000)$, where altitude is measured in metres (Scotto et al, 1996). The measure did not take into account cloud cover. We computed a lifetime time-weighted UV-residence index for each subject.

We calculated adjusted odds ratios, 95% confidence intervals, and tests for trend by means of multiple logistic regression models. Logistic regression was unconditional but included terms for the matching variables, i.e., sex and age. We used likelihood ratio tests for several parameters, and Wald tests for a simple parameter.

Stepwise-forward regression analysis was used to identify the strongest risk factors for CMM risk. Likelihood-ratio tests were used to select the most significant factors to be added to the initial logistic model. Variables with a $P$ value < 0.05 entered the model. The $P$ value for removal was > 0.10.

To evaluate associations of study variables with melanoma thickness, we carried out a case–case analysis using standard logistic regression methods with the occurrence of a lesion thicker than the median as the outcome.

We used the Wilcoxon (Mann–Whitney) rank-sum and Kruskal–Wallis rank tests for comparisons of 2 groups or more groups, respectively. We considered results significant if the $P$ value was <0.05.

Among controls, the hospital volunteers had higher number of hours of leisure sun exposure than the spouse/friend group and trauma patients ($P$ = 0.054). We repeated all analyses excluding the hospital volunteers, or including spouses/friends of the cases as the only control group, and we did not find major differences. The paper reports results obtained using the entire control group ($n$ = 179). We performed all analyses with the use of the Stata statistical package (Stata Corporation, College Station, TX–Release 6.0).

### RESULTS

### Subject characteristics

Details of personal characteristics of the 183 cases and 179 controls are summarised in Table 1. Hair colour, eye colour, skin colour, skin reaction after sun exposure, freckling, number of nevi, presence of DN, and sunburns with blistering were each strongly associated with CMM risk after adjustment for age and gender. When these variables were considered through forward stepwise regression analyses, presence of DN, skin colour, propensity to tan, and eye colour were retained with the matching variables. Using individuals with dark eyes as the comparison group, the relative odds of CMM was 1.8 (95% CI = 1.0–3.0) and 2.4 (95% CI = 1.1–5.2) for median and light eyes respectively ($P$ = 0.013, test for trend). Similarly, using subjects with dark skin as the comparison group, the CMM odds were 2.6 (95% CI = 0.9–7.2) and 4.1 (95% CI = 1.4–12.1) for medium and light skin colour respectively ($P$ = 0.009, test for trend). We obtained comparable results, with narrower confidence intervals, when subjects with medium skin colour were used as a reference group (data not shown). Subjects with medium and low propensity to tan after prolonged sun exposure had CMM relative odds of 0.9 (95% CI = 0.5–1.6) and 2.4 (95% CI = 1.1–5.0) when compared with subjects with high propensity to tan ($P$ = 0.027, test for trend). The relative odds for CMM due to presence of dysplastic nevi (OR = 4.4, 95% CI = 2.6–7.5), after adjustment for age and gender, did not substantially change after further adjustment for propensity to tan and skin and eye colour (OR = 4.2, 95% CI = 2.4–7.4).

In order to control for a possible bias due to frequent dermatological examinations, we re-analysed the association between DN and CMM risk adjusting the results for history of moles removed, or restricting the sample to thicker CMM lesions (above the median). We did not observe any important difference in the results (data not shown). Nevi and DN tend to disappear with age (Tucker et al, 1997). We repeated all analyses involving DN or nevi in the subset of subjects younger than 60. No major differences were observed. Results in the tables and text are based on the entire study group.

Men tended to have larger ORs than women. However, the tests for interaction for each risk factor with gender were not significant ($P$ = 0.12 for DN; $P$ = 0.65 for skin colour; $P$ = 0.17 for propensity to tan; and $P$ = 0.41 for eye colour).

### Sun exposure and CMM risk

Reported lifetime sun exposure during vacation between 11 am and 3 pm was comparable for cases and controls. Similarly, sun exposure due to vacation before the age of 18 years showed a relative odds close to one. This relative odds did not vary when adjusted for propensity to tan, skin colour, eye colour and presence of DN. The number of hours of sun exposure due to occupation was generally small in both cases and controls, and was not associated with CMM risk. Most subjects lived in the study area for their entire lifetime, and no difference was observed in the mean UVB-intensity index for cases and controls (median 101.8 RB units/year and 101.3 RB units/year for cases and controls, respectively, $P$ = 0.34). Only 7 cases and 4 controls had been in the tropics, with no association with CMM risk (OR = 1.6, 95% CI =

© 2001 Cancer Research Campaign

**Table 1** Melanoma risk by subject characteristics

| | Cases (*n* = 183) | Controls (*n* = 179) | OR | (95% CI) | Test for trend | OR | (95% CI) | Test for trend |
|---|---|---|---|---|---|---|---|---|
| | | | Adjusted for age and sex | | | Adjusted for multiple variables† | | |
| Sex | | | | | | | | |
| Males | 87 (47.5%) | 89 (49.7%) | | | | | | |
| Females | 96 (52.5%) | 90 (50.3%) | | | | | | |
| Mean age (±SD) | 48.5 ( ± 14.9) | 45.8 ( ± 13.6) | | | | | | |
| Hair colour | | | | | | | | |
| Black | 10 (5.6%) | 24 (13.4%) | 1.0 | – | | 1.0 | – | |
| Dark brown | 91 (50.6%) | 101 (56.4%) | 2.3 | (1.0–5.2) | | 1.7 | (0.7–4.4) | |
| Light/reddish brown | 54 (30.0%) | 43 (24.0%) | 3.3 | (1.4–7.9) | | 1.5 | (0.5–4.1) | |
| Blond | 23 (12.8%) | 11 (6.2%) | 5.4 | (1.9–15.7) | | 1.6 | (0.5–5.7) | |
| Red | 2 (1.1%) | 0 (0%) | – | – | < 0.001 | – | – | 0.640 |
| Eye colour* | | | | | | | | |
| Dark | 46 (25.1%) | 75 (42.1%) | 1.0 | – | | 1.0 | – | |
| Medium | 103 (56.3%) | 85 (47.8%) | 2.0 | (1.3–3.3) | | 1.8 | (1.0–3.0) | |
| Light | 34 (18.6%) | 18 (10.1%) | 3.0 | (1.5–6.0) | < 0.001 | 2.4 | (1.1–5.2) | 0.013 |
| Skin colour | | | | | | | | |
| Dark/olive | 6 (3.3%) | 29 (16.2%) | 1.0 | – | | 1.0 | – | |
| Medium | 73 (40.1%) | 92 (51.4%) | 3.4 | (1.3–8.7) | | 2.6 | (0.9–7.2) | |
| Light | 103 (56.7%) | 58 (32.4%) | 8.2 | (3.2–21.2) | < 0.001 | 4.1 | (1.4–12.1) | 0.009 |
| Freckles | 107 (59.1%) | 80 (45.2%) | 1.8 | (1.1–2.7) | | 1.3 | (0.8–2.1) | |
| Tanning ability to prolonged sun exposure | | | | | | | | |
| High | 35 (20.0%) | 61 (35.1%) | 1.0 | – | | 1.0 | – | |
| Medium | 80 (45.0%) | 87 (50.0%) | 1.5 | (0.9–2.6) | | 0.9 | (0.5–1.6) | |
| Low | 63 (35.4%) | 26 (14.9%) | 4.3 | (2.3–8.1) | < 0.001 | 2.4 | (1.1–5.0) | 0.027 |
| Skin response to 30 min in the sun | | | | | | | | |
| Tan without burn | 41 (22.4%) | 61 (34.1%) | 1.0 | – | | 1.0 | – | |
| Light/medium burn | 96 (52.5%) | 92 (51.4%) | 1.5 | (0.9–2.5) | | 1.2 | (0.7–2.1) | |
| Severe burn/blistering | 46 (25.1%) | 26 (14.5%) | 2.8 | (1.5–5.2) | 0.002 | 1.4 | (0.7–2.8) | 0.412 |
| Dysplastic nevi | 73 (39.9%) | 31 (17.3%) | 4.4 | (2.6–7.5) | | 4.2 | (2.4–7.4) | |
| Nevi number§ | | | | | | | | |
| 0–12 | 16 (10.0%) | 36 (21.2%) | 1.0 | – | | 1.0 | – | |
| 13–20 | 19 (11.9%) | 32 (18.8%) | 1.6 | (0.7–3.8) | | 1.8 | (0.7–4.6) | |
| 21–34 | 33 (20.6%) | 30 (17.7%) | 3.4 | (1.5–7.6) | | 3.0 | (1.2–7.6) | |
| 35–51 | 29 (18.1%) | 38 (22.4%) | 2.3 | (1.0–5.1) | | 1.7 | (0.7–4.4) | |
| 52–190 | 63 (39.4%) | 34 (20.0%) | 6.3 | (2.9–13.9) | < 0.001 | 3.4 | (1.3–8.9) | 0.027 |
| Lifetime number of sunburns with blisters | | | | | | | | |
| None | 149 (81.4%) | 152 (84.9%) | 1.0 | – | | 1.0 | – | |
| 1–5 | 19 (10.4%) | 23 (12.9%) | 0.9 | (0.5–1.7) | | 0.8 | (0.4–1.8) | |
| > 5 | 15 (8.2%) | 4 (2.2%) | 4.4 | (1.4–13.6) | 0.052 | 2.5 | (0.7–8.9) | 0.357 |

† Adjusted for age, sex, presence of dysplastic nevi, skin colour, eye colour, and tanning ability after prolonged sun exposure. * Dark: black or dark brown. Medium: light brown, brown-green, green, or blue-green. Light: light blue, dark blue or grey. § Nevi categories reflect quintile distribution in controls. Total number of subjects may vary across variables due to missing values.

0.4–6.6). Among leisure outdoor activities (such as, playing soccer, bicycling, fishing, hiking, sailing, etc.), swimming in the sea, lake, and/or pool was associated with a 2-fold CMM risk (30 cases and 38 controls, OR = 1.8, 95% CI = 0.9–3.4) after adjustment for eye colour, skin colour, propensity to tan and presence of dysplastic nevi.

### Use of sunscreens

Approximately 45% of cases and 43% of controls used sunscreens but most could not remember the protective factor, so this could not be assessed in the analysis. Ever/never sunscreen use did not substantially change the association between lifetime sun exposure during vacation and CMM risk after adjustment for age, gender, eye colour, skin colour, propensity to tan, and presence of DN (*P* value for the interaction between sun exposure and sunscreen use = 0.89). Use of sunscreen itself was not significantly associated with CMM risk or protection (OR = 1.2, 95% CI = 0.7–2.1), after adjustment for the strongest risk factors.

### Use of sunlamps and sunbeds

32 cases (17.6%) and 38 controls (21.2%), mostly with medium skin colour, high propensity to tan, and absence of DN, used sunlamps or sunbeds at least once during their lifetime. Sunlamp use was associated with a CMM relative odds of 1.3 (95% CI = 0.7–2.4), after adjustment for age, gender, skin colour, eye colour, propensity to tan and presence of DN. We did not find any association between number of sunlamp use and CMM risk (OR = 1.2, 95% CI = 0.6–2.6, for subjects who used sunlamps less than 10 times in their lifetime, and OR = 1.4, 95% CI = 0.5–3.6, for more than 10 times). Only a few subjects used sunlamps as part of a Photosensitising drug + UVA therapy (PUVA), so the association of PUVA therapy with CMM risk could not be evaluated.

 *© 2001 Cancer Research Campaign*

### Risk charts

We used forward stepwise regression analyses to identify the strongest CMM risk factors to be included in a parsimonious model. Overall, in order of relative significance, these were the presence of dysplastic nevi, skin colour, propensity to tan and eye colour. We constructed a flow chart to describe the relative risk associated with the combinations of 3-scale skin colour (dark-olive, medium, light), 3-scale eye colour (dark, medium, light), 2-scale propensity to tan (high/medium, low), and the presence of dysplastic nevi (yes/no), after adjustment for age and gender (Figure 1). We computed odds ratios for the combination of individual risk factors using the corresponding linear combinations of coefficients estimated by the logistic model. We used the categories at lowest risk for CMM (i.e. no DN, dark-olive skin colour, high/medium propensity to tan, dark eye colour) as reference category. An analysis using medium skin colour as a reference point would provide relative-risk estimates with narrower confidence intervals and with the same rank order. We decided to present results using the dark skin category as a reference point for an easier comprehension. Of note, when analyses were restricted to subjects younger than 60, the combination of presence of DN, light skin colour, low propensity to tan and light eye colour produced an OR = 215 (95% CI = 38–1217).

### CMM thickness

Thickness of melanoma lesions varied from 0.1 to 8.5 mm. Melanoma thickness increased with age, but the association was not statistically significant after adjustment for gender, eye colour, skin colour, propensity to tan and presence of DN ($P$ = 0.15). When cases were categorised into 2 groups by CMM thickness, above and below the median (0.9 mm), light skin colour (OR = 3.6, 95% CI = 1.7–7.6), low propensity to tan (OR = 2.7, 95% CI = 1.3–5.8), and history of sunburns with blistering (OR = 3.3, 95% CI = 1.3–8.2) were significantly associated with CMM thickness (Table 2), after adjustment for age and gender. Conversely, presence of dysplastic nevi (OR = 0.6, 95% CI = 0.3–1.2) and high number of common nevi (OR = 0.5, 95%

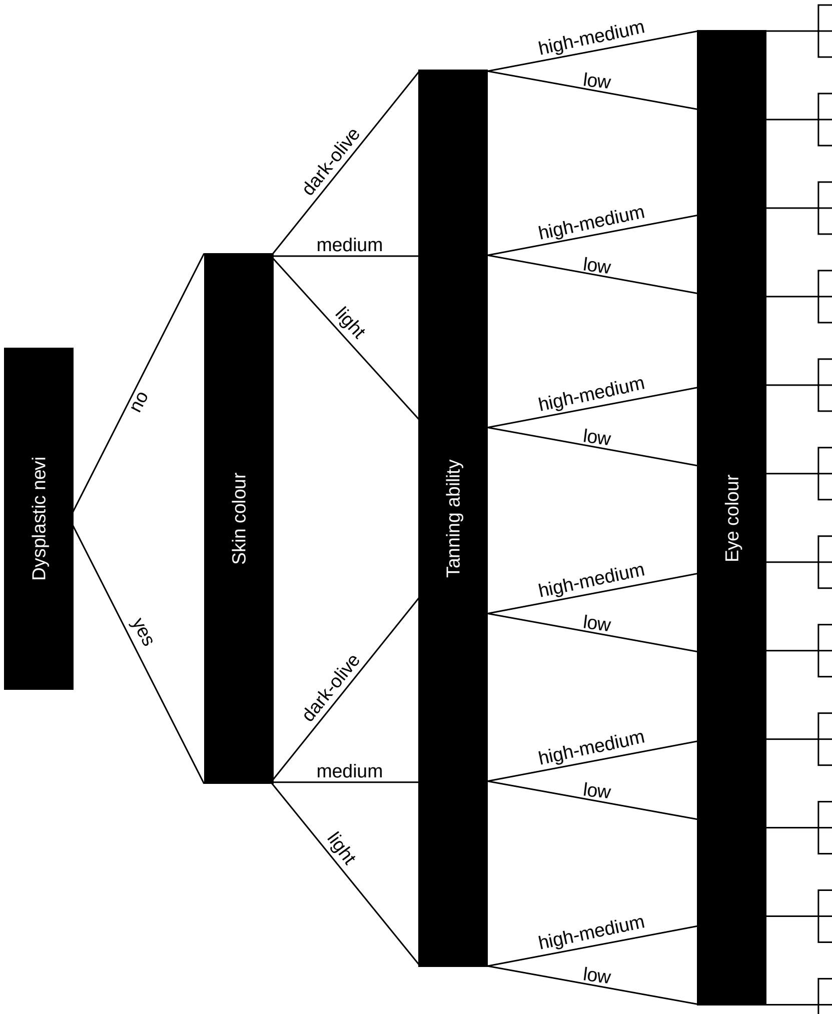

| Dysplastic nevi | Skin colour | Tanning ability | Eye colour | N. of subjects | Modeled OR | (95% CI) |
|---|---|---|---|---|---|---|
| no | dark-olive | high-medium | dark | (n = 12) | 1.0 | – |
| no | dark-olive | high-medium | medium | (n = 14) | 1.8 | (1.0–3.0) |
| no | dark-olive | high-medium | light | (n = 3) | 2.4* | (1.1–5.1)* |
| no | dark-olive | low | dark | (n = 0) | 2.6* | (1.4–4.7)* |
| no | dark-olive | low | medium | (n = 0) | 4.5* | (2.0–10.3)* |
| no | dark-olive | low | light | (n = 0) | 6.1* | (2.3–16.7)* |
| no | medium | high-medium | dark | (n = 31) | 2.4 | (0.9–6.6) |
| no | medium | high-medium | medium | (n = 36) | 4.3 | (1.4–13.3) |
| no | medium | high-medium | light | (n = 10) | 5.8 | (1.7–20.3) |
| no | medium | low | dark | (n = 4) | 6.3 | (2.0–19.9) |
| no | medium | low | medium | (n = 5) | 11.1 | (3.1–39.7) |
| no | medium | low | light | (n = 1) | 15.0* | (3.7–60.2)* |
| no | light | high-medium | dark | (n = 6) | 3.8 | (1.4–10.7) |
| no | light | high-medium | medium | (n = 28) | 7.0 | (2.2–20.5) |
| no | light | high-medium | light | (n = 8) | 9.0 | (2.6–31.4) |
| no | light | low | dark | (n = 10) | 9.9 | (3.3–29.5) |
| no | light | low | medium | (n = 21) | 17.3 | (5.3–56.6) |
| no | light | low | light | (n = 2) | 23.4* | (6.3–86.7)* |
| yes | dark-olive | high-medium | dark | (n = 1) | 4.2* | (2.4–7.4)* |
| yes | dark-olive | high-medium | medium | (n = 1) | 7.4* | (3.3–16.7)* |
| yes | dark-olive | high-medium | light | (n = 0) | 10.0* | (3.8–26.5)* |
| yes | dark-olive | low | dark | (n = 0) | 10.9* | (4.6–25.5)* |
| yes | dark-olive | low | medium | (n = 1) | 19.1* | (6.7–54.3)* |
| yes | dark-olive | low | light | (n = 0) | 25.8* | (7.9–84.2)* |
| yes | medium | high-medium | dark | (n = 17) | 10.3 | (3.4–30.8) |
| yes | medium | high-medium | medium | (n = 17) | 18.0 | (5.2–62.6) |
| yes | medium | high-medium | light | (n = 6) | 24.4 | (6.4–93.8) |
| yes | medium | low | dark | (n = 4) | 26.5* | (7.6–92.9)* |
| yes | medium | low | medium | (n = 1) | 46.6* | (11.6–187.2)* |
| yes | medium | low | light | (n = 1) | 63.1* | (14.2–279.3)* |
| yes | light | high-medium | dark | (n = 8) | 16.1 | (5.2–49.9) |
| yes | light | high-medium | medium | (n = 16) | 28.2 | (8.2–97.1) |
| yes | light | high-medium | light | (n = 3) | 38.1* | (10.0–146.0)* |
| yes | light | low | dark | (n = 2) | 41.6 | (12.4–138.8) |
| yes | light | low | medium | (n = 16) | 72.8 | (19.6–270.5) |
| yes | light | low | light | (n = 8) | 98.5* | (23.8–406.9)* |

**Figure 1** Relative risk of melanoma due to the combination of multiple risk factors
*Results extrapolated from logistic regression analysis

*© 2001 Cancer Research Campaign* 

**Table 2** Risk of thick melanoma lesions by case characteristics

| | Melanoma thickness | | OR | (95% CI) |
|---|---|---|---|---|
| | **< median (*n* = 75)** | **≥ median (*n* = 76)** | | |
| Hair colour | | | | |
| Black/dark brown | 47 (62.7%) | 35 (47.3%) | 1.0 | – |
| Light/reddish brown | 20 (26.7%) | 26 (35.1%) | 1.9 | (0.9–4.2) |
| Blond/red | 8 (10.7%) | 13 (17.6%) | 2.5 | (0.8–7.3) |
| Eye colour* | | | | |
| Dark | 22 (29.3%) | 17 (22.4%) | 1.0 | – |
| Medium | 39 (52.0%) | 46 (60.5%) | 1.5 | (0.7–3.3) |
| Light | 14 (18.7%) | 13 (17.1%) | 1.2 | (0.5–3.3) |
| Skin colour | | | | |
| Dark-olive/medium | 42 (56.0%) | 22 (29.3%) | 1.0 | – |
| Light | 33 (44.0%) | 53 (70.7%) | 3.6 | (1.7–7.6) |
| Freckles | 44 (58.7%) | 46 (62.2%) | 1.1 | (0.6–2.3) |
| Tanning ability to prolonged sun exposure | | | | |
| High/medium | 53 (72.6%) | 39 (53.4%) | 1.0 | – |
| Low | 20 (27.4%) | 34 (46.6%) | 2.7 | (1.3–5.8) |
| Skin response to 30 min in the sun | | | | |
| Tan without burn | 22 (29.3%) | 13 (17.1%) | 1.0 | – |
| Light/medium burn | 34 (45.3%) | 46 (60.5%) | 2.3 | (1.0–5.4) |
| Severe burn/blistering | 19 (25.3%) | 17 (22.4%) | 1.7 | (0.6–4.5) |
| Dysplastic nevi | 34 (45.3%) | 25 (32.9%) | 0.6 | (0.3–1.2) |
| Nevi number § | | | | |
| 0–27 | 18 (28.6%) | 26 (36.1%) | 1.0 | – |
| 27–56 | 18 (28.6%) | 27 (37.5%) | 1.1 | (0.4–2.6) |
| 56–190 | 27 (42.9%) | 19 (26.4%) | 0.5 | (0.2–1.2) |
| Sunburns with blisters | 8 (10.7%) | 21 (27.6%) | 3.3 | (1.3–8.2) |
| Lifetime exposure to sun during vacation † | | | | |
| 0–150 hours | 21 (29.6%) | 32 (44.4%) | 1.0 | – |
| 150–1,500 hours | 24 (33.8%) | 22 (30.6%) | 0.7 | (0.3–1.5) |
| 1,500–18,500 hours | 26 (36.6%) | 18 (25.0%) | 0.5 | (0.2–1.2) |
| Exposure to sun during vacation in childhood ‡ | | | | |
| None | 27 (38.0%) | 41 (56.9%) | 1.0 | – |
| 10–900 hours | 20 (28.2%) | 21 (29.2%) | 0.7 | (0.3–1.7) |
| 900–8,200 hours | 24 (33.8%) | 10 (13.9%) | 0.3 | (0.1–0.7) |

* Dark: black or dark brown. Medium: light brown, brown-green, green, or blue-green. Light: light blue, dark blue or grey.
§ Nevi categories reflect tertile distribution in all cases. † Hours of lifetime outdoor exposure between 11 AM and 3 PM.
‡ Hours of outdoor exposure between 11 AM and 3 PM under age 18. Odds ratios and confidence intervals adjusted for age and sex in multivariable logistic regression analysis. Total number of subjects may vary across variables due to missing values.

CI = 0.2–1.7) were negatively, although not significantly, associated with melanoma thickness. Similarly, the more hours of sun exposure during vacation, the thinner the CMM lesions were, particularly for sun exposure during childhood (OR for the highest exposure category = 0.3, 95% CI = 0.1–0.7; $P$ = 0.01, test for trend). Results reported in Table 2 did not substantially change after adjustment for skin colour, propensity to tan, and presence of DN, in addition to gender and age.

### DISCUSSION

Melanoma incidence rates are rapidly increasing in Mediterranean countries. Because advanced melanoma is still largely incurable, early detection and identification of factors associated with the development and progression of the disease are of great importance. We found that presence of dysplastic (or atypical) nevi, light skin colour, light eye colour, and low propensity to tan are the strongest risk factors for a population from north-eastern Italy. Light skin colour, light eye colour and low propensity to tan, singularly or in combination, contributed strongly to CMM risk, even after adjustment for the presence of DN. There was no interaction between each phenotypic characteristic and presence of DN in the association with CMM risk. A recent study conducted in Italy (Naldi et al, 2000a) identified similar risk factors, along with history of sunburns before age 15 years, and presence of solar lentigines. Precise history of sunburns is difficult to recall, and identification of solar lentigines, as distinct from freckles, is difficult and can vary among dermatologists. In addition, the diagnosis of DN and nevi is more difficult in older subjects. We attempted to minimise assessment variability by (1) having only one dermatologist to perform the skin examinations, (2) verifying the results in subjects younger than 60, and (3) having one oncologist to assess all DN and nevi diagnoses on photographs. Recognition and classification of DN from photographs have been shown to be accurate and reproducible (Hartge et al, 1995).

Studies in Scotland (MacKie et al, 1989) and West Germany (Garbe et al, 1994) showed how melanoma risk was affected by a combination of multiple risk factors. Our chart describes how the

 *© 2001 Cancer Research Campaign*

combination of DN, propensity to tan, skin colour, and eye colour can affect CMM relative risk in an Italian population. We used the logistic model to predict risk estimates for combinations of several factors. Since the analyses were based on relatively small numbers of cases and controls, results should be used with caution. This is particularly true for risk-factor combinations that were not present among cases and/or controls, because of their rarity in the general Mediterranean population, and/or in subjects affected by CMM. In fact, in this study, there was only one subject with dark-olive skin and low propensity to tan, and there were only 3 subjects with DN and dark-olive skin.

In the top part of the chart, CMM risk is based on the combination of factors that can be immediately recognised by both patients and clinical personnel, thus allowing for an easy identification of at-risk people that need particular attention. The bottom part adds to the phenotypic characteristics the contribution of the presence of DN, which requires more skills and experience to assess. CMM personal risk may increase 70 times or more if combined with the factors in the top part of the chart.

Interestingly, subjects with light skin colour and low propensity to tan and those who experienced sunburns with blistering had generally thicker CMM lesions. Such subjects may not be aware that they are at high risk and defer seeking medical attention until their lesion is large. We cannot verify this hypothesis, but we feel these subjects should be the targets for public education campaigns for early detection or prevention of melanoma in Mediterranean countries. Subjects with DN or many nevi are possibly more aware of their at-risk condition, and in fact, had generally thinner CMM lesions. In addition, subjects who spend long periods of time under the sun in bathing suits are more likely to be observed by other people, who may notice and call attention to suspicious skin lesions at an early stage. In fact, high number of hours of sun exposure, particularly during childhood, was associated with thinner CMM lesions.

The amount of lifetime sun exposure is notoriously difficult to assess, so imprecision in reporting hours of exposure as well as recall bias are possible. The study participants were all from the same area. In addition, the great majority of the controls were spouses or partners of the cases, thus likely sharing with cases similar sun and other environmental exposures. Finally, most study subjects lived in the area all their life, had very similar UVB index, and only a few experienced trips to the tropics. Consequently, variability in sun exposure was small in this population. In Italy, a positive association (Rosso et al, 1998; Zanetti et al, 1999) and a lack of association (Cristofolini et al, 1987; Carli et al, 1995) between sun exposure and CMM risk have been found. Our goal was not to examine the association between sun exposure and melanoma, but rather to identify subjects at high risk because of inherited risk factors, independently of their amount of sun exposure. Thus, the similarity between cases and controls in regard to environmental exposures was appropriate for our goal.

Among other factors, sunscreen use was not associated with melanoma risk as shown in previous studies around the world (Elwood and Gallagher, 1999; La Vecchia, 1999) and in the Italian population (Naldi et al, 2000b). Use of sunbeds or sunlamps was higher than found in other Italian studies (Naldi et al, 2000c), but was not associated with CMM risk.

In conclusion, screening for melanoma is not yet common in Italy and other Mediterranean countries. The magnitude of risk associated with the combination of DN, and/or light eye, light skin, and low propensity to tan shows the need of preventive advice against melanoma in these populations.

### ACKNOWLEDGEMENTS

This study was supported by a grant (CA 65558–01A2) from the National Institute of Health to MT Landi, and by the contract N02-CP-91026 of the Genetic Epidemiology Branch, NCI.

We are indebted to Ken Kraemer MD for his helpful comments and encouragement. We are also very grateful to the study subjects and personnel of the Bufalini Hospital for their participation.

### REFERENCES

Bliss JM, Ford D, Swerdlow AJ, Armstrong BK, Cristofolini M, Elwood JM, Green A, Holly EA, Mack T and MacKie RM (1995) Risk of cutaneous melanoma associated with pigmentation characteristics and freckling: systematic overview of 10 case-control studies. The International Melanoma Analysis Group (IMAGE). *Int J Cancer* **62**: 367–376

Calista D, Goldstein AM and Landi MT (2000) Melanoma familial aggregation in north-eastern italy. *J Invest Dermatol* **115**: 764–765

Carli P, Biggeri A and Giannotti B (1995) Malignant melanoma in Italy: risks associated with common and clinically atypical melanocytic nevi. *J Am Acad Dermatol* **32**: 734–739

Cristofolini M, Franceschi S, Tasin L, Zumiani G, Piscioli F, Talamini R and La Vecchia C (1987) Risk factors for cutaneous malignant melanoma in a northern Italian population. *Int J Cancer* **39**: 150–154

Elwood JM and Gallagher RP (1999) More about: sunscreen use, wearing clothes, and number of nevi in 6- to 7-year-old European children. *J Natl Cancer Inst* **91**: 1164–1166

Espinosa Arranz, J, Sanchez Hernandez JJ, Bravo Fernandez P, Gonzalez-Baron M, Zamora Aunon P, Espinosa Arranz E, Jalon Lopez JI and Ordonez Gallego A (1999) Cutaneous malignant melanoma and sun exposure in Spain. *Melanoma Res* **9**: 199–205

Ferlay J, Bray S, Pisani P and Parkin DM (2001) *GLOBOCAN 2000: Cancer incidence, mortality and prevalence worldwide, Version 1.0*. CancerBase No. 5. IARCPress: Lyon. Limited version available from: URL: http://www-dep.iarc.fr/globocan/globocan.htm

Garbe C, Buttner P, Weiss J, Soyer HP, Stocker U, Kruger S, Roser M, Weckbecker J, Panizzon R and Bahmer F (1994) Risk factors for developing cutaneous melanoma and criteria for identifying persons at risk: multicenter case-control study of the Central Malignant Melanoma Registry of the German Dermatological Society. *J Invest Dermatol* **102**: 695–699

Goldstein AM and Tucker MA (1995) Genetic epidemiology of familial melanoma. *Dermatol Clin* **13**: 605–612

Greene MA and Fraumeni JF Jr (1979) The hereditary variant of malignant. melanoma. In *Human malignant melanoma*, Clark WHJr, Goldman LI, Mastrangelo LJ (eds) pp 139–166. Grune and Stratton: New York

Hartge P, Holly EA, Halpern A, Sagebiel R, Guerry D, Elder D, Clark W, Hanson L, Harrison C and Tarone R (1995) Recognition and classification of clinically dysplastic nevi from photographs: a study of interobserver variation. *Cancer Epidemiol Biomarkers Prev* **4**: 37–40

Jemal A, Devesa SS, Fears TR and Hartge P (2000) Cancer surveillance series: changing patterns of cutaneous malignant melanoma mortality rates among whites in the United States. *J Natl Cancer Inst* **92**: 811–818

La Vecchia C (1999) Sunscreens and the risk of cutaneous malignant melanoma. *Eur J Cancer Prev* **8**: 267–269

Landi MT, Calista D, Landi G, Bernucci I, Bertazzi PA, Clark WHJ, Goldstein AM and Tucker MA (1999) Clinical characteristics of 20 Italian melanoma-prone families. *Arch Dermatol* **135**: 1554–1555

MacKie RM, Freudenberger T and Aitchison TC (1989) Personal risk-factor chart for cutaneous melanoma. *Lancet* **2**: 487–490

Muir CS, Waterhouse J, Mack T, Powell J and Whelan SL (1987) *Cancer incidence in five continents*. International Agency for Research on Cancer: Lyon

Naldi L, Lorenzo IG, Parazzini F, Gallus S and La Vecchia C (2000a) Pigmentary traits, modalities of sun reaction, history of sunburns, and melanocytic nevi as risk factors for cutaneous malignant melanoma in the Italian population: results of a collaborative case-control study. *Cancer* **88**: 2703–2710

Naldi L, Gallus S, Imberti GL, Cainelli T, Negri E and La Vecchia C (2000b) Sunscreens and cutaneous malignant melanoma: an Italian case-control study. *Int J Cancer* **86**: 879–882

Naldi L, Gallus S, Imberti GL, Cainelli T, Negri E and La Vecchia C (2000c) Sunlamps and sunbeds and the risk of cutaneous melanoma. Italian Group for Epidemiological Research in Dermatology. *Eur J Cancer Prev* **9**: 133–134

*© 2001 Cancer Research Campaign* 

Parkin DM, Muir CS, Whelan SL, Gao YT, Ferlay J and Powell J (1992) *Cancer incidence in five continents*. International Agency for Research on Cancer: Lyon

Rodenas JM, Delgado-Rodriguez M, Herranz MT, Tercedor J and Serrano S (1996) Sun exposure, pigmentary traits, and risk of cutaneous malignant melanoma: a case-control study in a Mediterranean population. *Cancer Causes Control* **7**: 275–283

Rosso S, Zanetti R, Pippione M and Sancho-Garnier H (1998) Parallel risk assessment of melanoma and basal cell carcinoma: skin characteristics and sun exposure. *Melanoma Res* **8**: 573–583

Scotto J, Fears TR and Fraumeni JF Jr. (1996) Solar radiation. In *Cancer epidemiology and prevention*, Schottenfeld D, Fraumeni JF Jr. (eds) pp 355–372. Oxford University Press: New York

Tucker MA, Halpern A, Holly EA, Hartge P, Elder DE, Sagebiel RW, Guerry D and Clark WHJ (1997) Clinically recognized dysplastic nevi. A central risk factor for cutaneous melanoma. *JAMA* **277**: 1439–1444

Vinceti M, Bergomi M, Borciani N, Serra L and Vivoli G (1999) Rising melanoma incidence in an Italian community from 1986 to 1997. *Melanoma Res* **9**: 97–103

Zanetti R, Rosso S, Colonna S, Martina G and Paudice A (1988) Studio caso-controllo sul melanoma maligno cutaneo in provincia di Torino [Case-control study on malignant skin melanoma in the Turin province]. *G Ital Dermatol Venereol* **123**: 461–468

Zanetti R, Gafa L, Franceschi S, Pippione M and Rosso S (1999) Estimate of the proportion of skin tumors attributable to sun exposure in 3 Italian populations. *Epidemiol Prev* **23**: 416–422

*© 2001 Cancer Research Campaign*
